# Mapping and visualization of global research progress on deubiquitinases in ovarian cancer: a bibliometric analysis

**DOI:** 10.3389/fphar.2024.1445037

**Published:** 2024-09-12

**Authors:** Fang Qiu, Yuntong Li, Lile Zhou, Yingli Wu, Yunzhao Wu, Zhilei Fan, Yingying Wang, Dongjun Qin, Chaoqun Li

**Affiliations:** ^1^ Department of Burn and Plastic Surgery, Shenzhen Longhua District Central Hospital, Shenzhen, Guangdong, China; ^2^ Faculty of Geosciences and Environmental Engineering, Southwest Jiaotong University, Chengdu, Sichuan, China; ^3^ Hongqiao International Institute of Medicine, Shanghai Tongren Hospital/Faculty of Basic Medicine, Department of Pathophysiology, Key Laboratory of Cell Differentiation and Apoptosis of the Chinese Ministry of Education, Shanghai Jiao Tong University School of Medicine, Shanghai, China; ^4^ Yusuf Hamied Department of Chemistry, University of Cambridge, London, United Kingdom; ^5^ School of Public Health, Fudan University, Shanghai, China; ^6^ Department of Histology and Embryology, Shanghai Key Laboratory of Cell Engineering, Naval Medical University, Shanghai, China

**Keywords:** ovarian cancer, deubiquitinating enzyme, bibliometric analysis, biologic role, systematic review

## Abstract

**Background:**

Ovarian cancer is a highly aggressive malignancy with limited therapeutic options and a poor prognosis. Deubiquitinating enzymes (DUBs) have emerged as critical regulators of protein ubiquitination and proteasomal degradation, influencing various cellular processes relevant to cancer pathogenesis. In this study, the research progress between ovarian cancer and DUBs was mapped and visualized using bibliometrics, and the expression patterns and biological roles of DUBs in ovarian cancer were summarized.

**Methods:**

Studies related to DUBs in ovarian cancer were extracted from the Web of Science Core Collection (WoSCC) database. VOSviewer 1.6.20, CiteSpace 6.3.R1, and R4.3.3 were used for bibliometric analysis and visualization.

**Results:**

For analysis 243 articles were included in this study. The number of publications on DUBs in ovarian cancer has gradually increased each year. China, the United States, and the United Kingdom are at the center of this field of research. The Johns Hopkins University, Genentech, and Roche Holding are the main research institutions. David Komander, Zhihua Liu, and Richard Roden are the top authors in this field. The top five journals with the largest publication volumes in this field are *Biochemical and Biophysical Research Communications*, *Journal of Biological Chemistry*, *PLOS One*, *Nature Communications*, and *Oncotarget*. Keyword burst analysis identified five research areas: “deubiquitinating enzyme,” “expression,” “activation,” “degradation,” and “ubiquitin.” In addition, we summarized the expression profiles and biological roles of DUBs in ovarian cancer, highlighting their roles in tumor initiation, growth, chemoresistance, and metastasis.

**Conclusion:**

An overview of the research progress is provided in this study on DUBs in ovarian cancer over the last three decades. It offers insight into the most cited papers and authors, core journals, and identified new trends.

## Introduction

Ovarian cancer, which is the fifth most prevalent cancer among women, significantly contributes to global cancer-related mortalities in women (Siegel et al., 2023). Due to the non-specific or subtle symptoms associated with this disease, early detection and diagnosis remain challenging. Consequently, it is frequently diagnosed at advanced stages, leading to undesirable outcomes. Previous studies have identified various risk factors for ovarian cancer, including family history, age, obesity, genetic mutations, and early onset of menstruation (Wang et al., 2023a; Sung et al., 2023; Sandvei et al., 2023; Matan et al., 2022; Fortner et al., 2019; Arora et al., 2024). However, more efforts are still required to establish effective screening strategies, such as protein biomarkers, for the early diagnosis of ovarian cancer.

Post-translational modification plays an important role in regulating target protein activity, stability, interaction, and/or localization (Singh and Ostwal, 2019; Lee et al., 2023; Wang et al., 2022a; Li et al., 2023). Acetylation, sumoylation, ubiquitination, and phosphorylation are the most common types of protein post-translational modification (Wang et al., 2014a). Specifically, ubiquitination is a process in which an ubiquitin (Ub) protein, or a chain of Ub proteins, is covalently attached to the target substrate, ultimately leading to the proteasomal degradation or localization alteration of the target protein (Damgaard, 2021). This process can be reversed by deubiquitinases (DUBs), which cleave ubiquitin from targeted proteins (Snyder and Silva, 2021). The dynamic balance between ubiquitination and deubiquitination plays critical roles in biological activities, such as cell-signaling transduction, apoptosis, and drug resistance. To date, six classes of DUBs have been identified, namely, ovarian tumor proteases (OTUs), ubiquitin-specific proteases (USPs), ubiquitin C-terminal hydrolases (UCHs), and Josephin domain-containing proteins, MINDYs, and JAMMs (Harrigan et al., 2018). Among them, USPs form the largest family of DUBs. Accumulating evidence suggests that the dysregulation of USPs is involved in various diseases, including cancer. For example, we previously found that targeting USP47 could decrease tyrosine kinase inhibitor resistance and eradicate leukemia stem/progenitor cells in chronic myelogenous leukemia (Lei et al., 2021a). We and others have suggested that USP7 plays essential biological roles in the pathogenesis of multiple myelomas (Jing et al., 2018; Wang et al., 2022b; Chauhan et al., 2012). Importantly, USP7 has also been revealed as a promising target for ovarian cancer treatment (Ma and Yu, 2016; Zhang et al., 2016; Qin et al., 2016). Thus, DUBs, especially USPs, may serve as promising biomarkers for the early detection and diagnosis of ovarian cancer.

In this study, we performed a bibliometric analysis of the scientific articles published on DUBs in ovarian cancer to evaluate the study trends on this topic. Although several bibliometric analyses have been published on various topics in ovarian cancer (Song et al., 2024; Lin et al., 2024; Meng et al., 2024; Wang et al., 2024; Leng et al., 2023; Giles et al., 2023; Duan et al., 2023; Liu et al., 2023a), this is the first study to identify the most influential literature in this field. We also summarized the expression and biological roles of DUBs in ovarian cancer and explored their potential as biomarkers.

## Methods

### Data sources and search strategy

The literature search was conducted to retrieve related articles from inception to May 2024 from the Web of Science Core Collection (WoSCC). The search strategy is presented in Supplementary Table S1. This study included only “articles” and considered only documents written in English. As all data were obtained from a public database, ethical declarations or approvals are not applicable.

### Data analysis and visualization

We extracted relevant data from the retrieved literature titles and used Microsoft Excel 16.0 to identify and calculate bibliometric parameters. These metrics cover key aspects of publications, including the number of publications per year, citation frequency, average citation frequency, journal title, journal impact factor, country/region of publication, publishing organization, and authors.

The visualization and analysis process involved the use of three powerful bibliometric analysis tools to fully analyze the academic data: VOSviewer (version 1.6.20), CiteSpace (version 6.3.R1), and R4.3.3. VOSviewer is a versatile software tool that plays a key role in mapping institutional collaborations, co-authorships, citations, and co-citations (van Eck and Waltman, 2010). It was used for keyword co-occurrence analysis. CiteSpace 6.3.R1 was used for keyword emergence detection and co-occurrence analysis, with the parameters set to time slicing: from January 1996 to May 2024 (research in this field was originally published in 1996). The time slicing was set to 1 year, and the node types were set to keywords. When nodes are keywords, the threshold (top N per segment) was set to 5, and pruning was set to the pathfinder + pruning merged network. Based on the parameter settings for each node, a visual analysis was performed to generate a timeline graph of deubiquitinating enzymes with keywords in the field of ovarian cancer research.

## Results

### Overview of the main information

**FIGURE 1:**
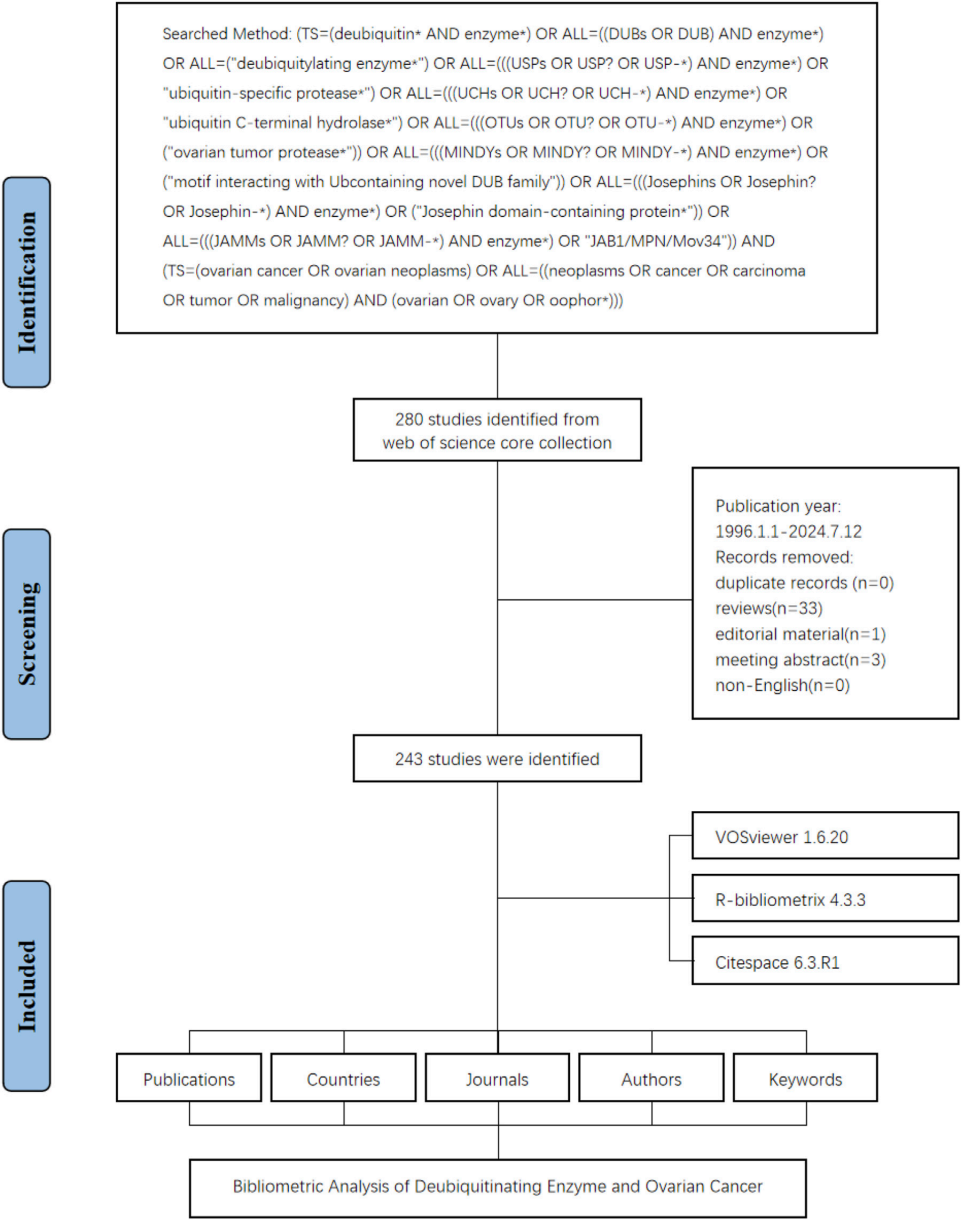
Study flowchart.

**FIGURE 2:**
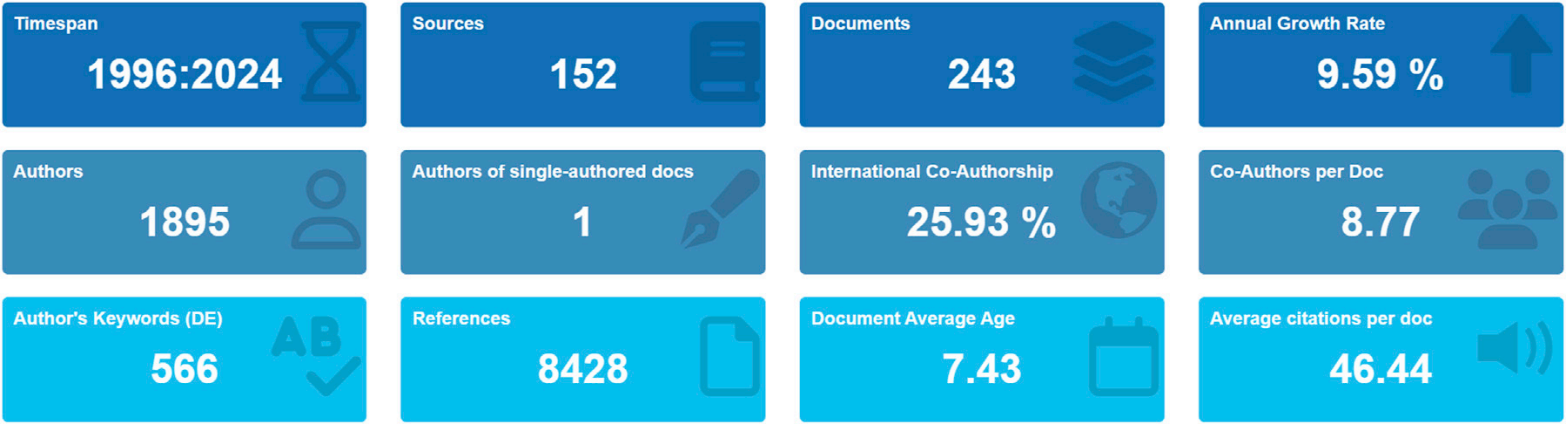
Overview of the main information.

The study flowchart is presented in Figure 1. A total of 243 articles were identified in this study on DUBs in ovarian cancer over the last three decades. Our investigation showed that 1,895 authors from 926 institutions across 135 countries contributed to the production of these 243 manuscripts. These works were published in 152 journals, citing 8,428 references, with an average of 46.44 citations per article (Figure 2).

### Annual publication trend

**FIGURE 3:**
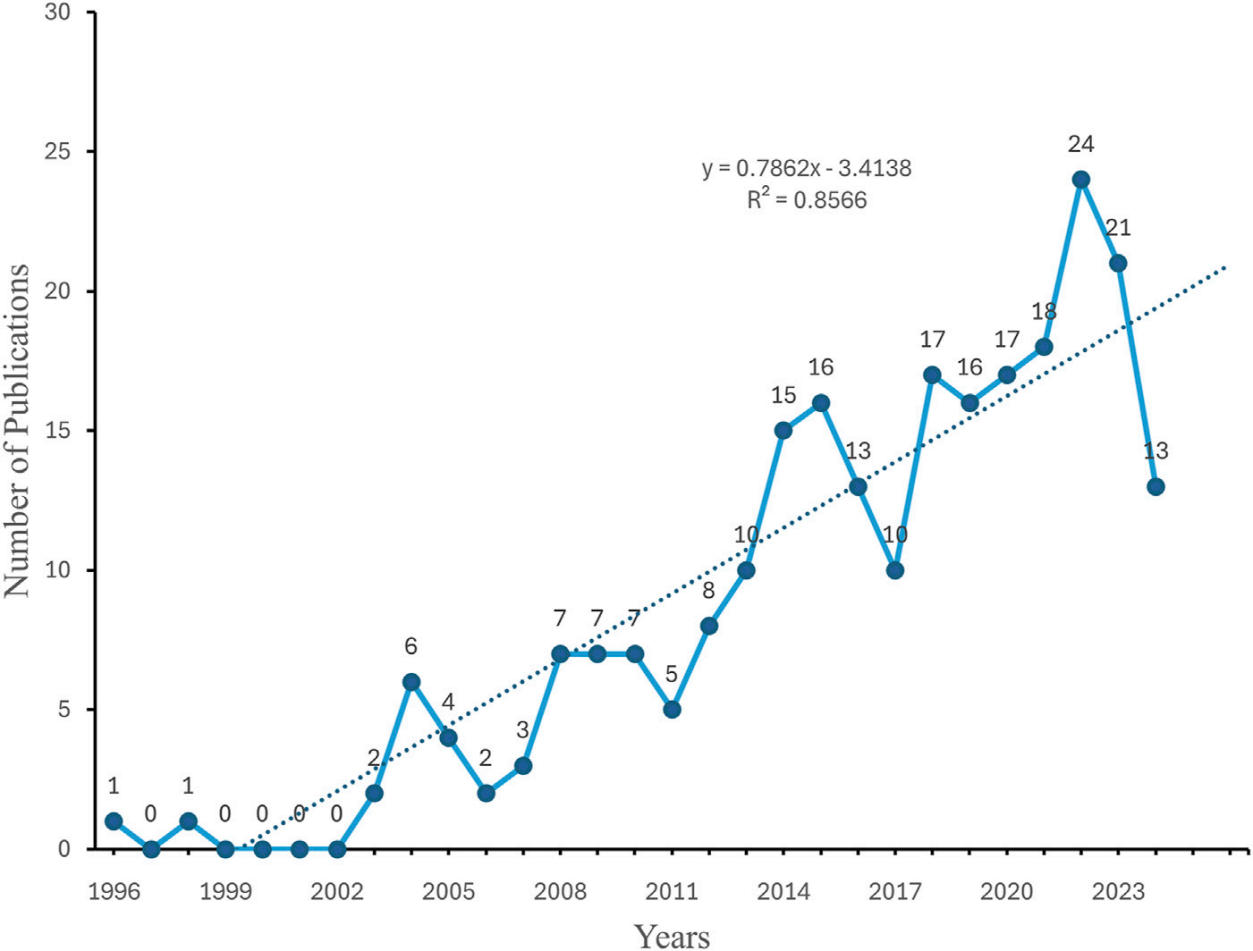
Annual number of publications on deubiquitinating enzymes and ovarian cancer.

To gain insight into the evolution of related research in this field, we examined the annual publication trends. The study period exhibited a discernible upward trajectory in annual publications, particularly since 2003. The change in cumulative publications over time follows the trend line equation y = 0.7,862 × −3.4,138, with a correlation coefficient of 0.8566 and an annual growth rate of 9.59%. Additionally, 2022 witnessed the highest number of publications, accounting for 9.88% of the total (Figures 2, 3).

### Analysis of countries

**FIGURE 4:**
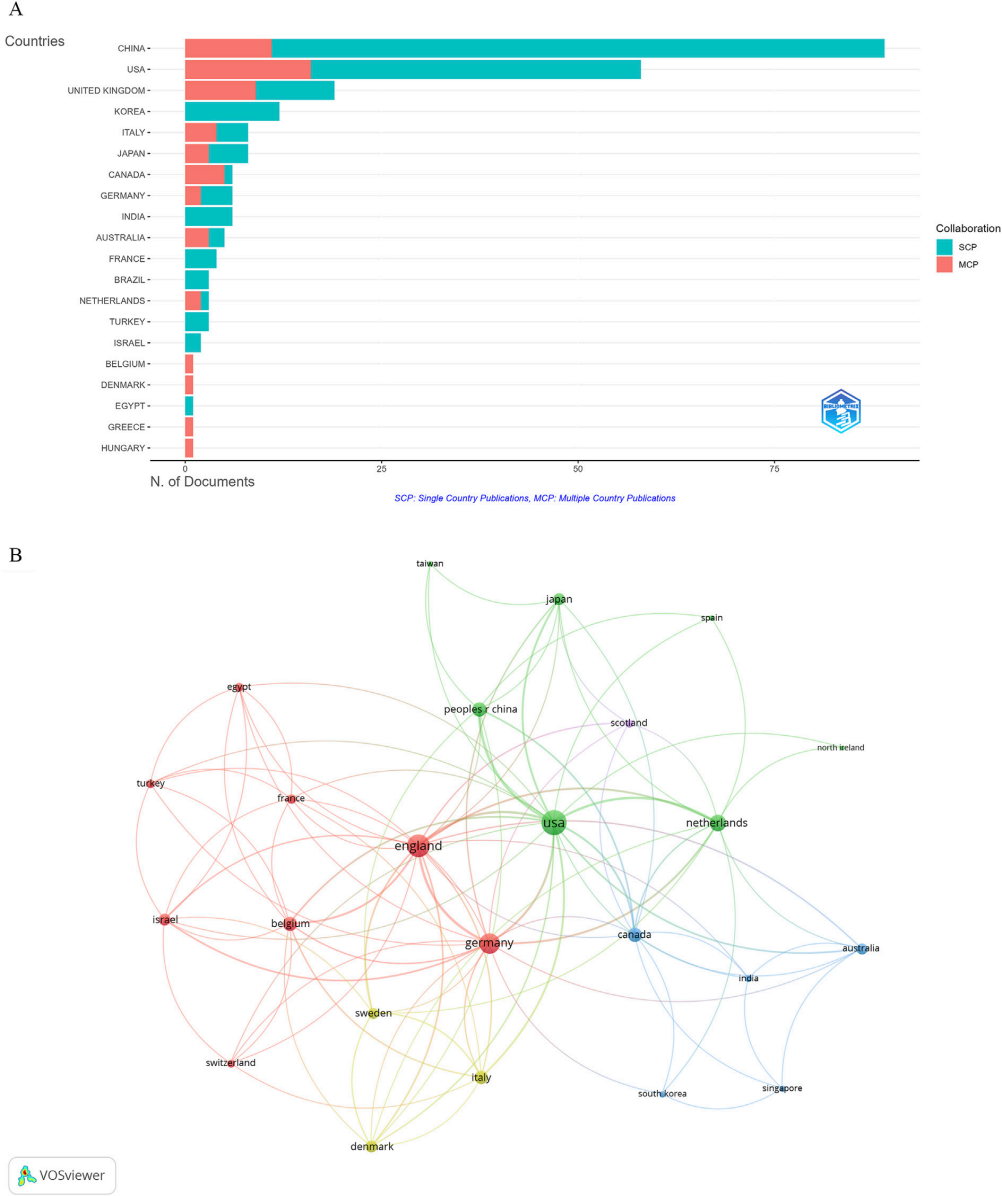
Visualization of countries. **(A)** Publications by country. **(B)** International collaboration network.

The identified publications came from 135 countries, with China leading in the number of studies (89 publications), constituting 36.62% of all documents. Other top contributors included the United States of America (58 publications), the United Kingdom (19 publications), Korea (12 publications), Japan (8 publications), and Italy (8 publications) (Figure 4A; Table 1). Despite China having the highest number of articles, the United States of America, France, and the United Kingdom had the highest average citations, that is, 96.9, 89.8, and 87.6, respectively. In addition, the collaboration among countries was visualized using VOSviewer. As shown in Figure 4B, the United States, the United Kingdom, and Germany were the top three countries with the strongest international collaboration network.

**TABLE 1:** Publication and citation profiles of the top 10 countries.

Country	Articles	Freq	MCP_Ratio	TP	TP_rank	TC	TC_rank	Average citations
China	89	0.366	0.124	302	1	1,595	3	17.9
United States of America	58	0.239	0.276	283	2	5,619	1	96.9
United Kingdom	19	0.078	0.474	40	4	1,664	2	87.6
Korea	12	0.049	0.000	36	5	179	9	14.9
Italy	8	0.033	0.500	46	3	157	10	19.6
Japan	8	0.033	0.375	33	6	234	8	29.2
Canada	6	0.025	0.833	26	9	237	7	39.5
Germany	6	0.025	0.333	30	7	70	13	11.7
India	6	0.025	0.000	22	10	86	12	14.3
Australia	5	0.021	0.600	29	8	284	6	56.8

Note(s): Articles, publications of corresponding authors only; Freq, frequency of total publications; MCP_Ratio, proportion of multiple country publications; TP, total publications; TP_rank, rank of total publications; TC, total citations; TC_rank, rank of total citations; Average citations, average number of citations per publication.

### Analysis of institutions

**FIGURE 5:**
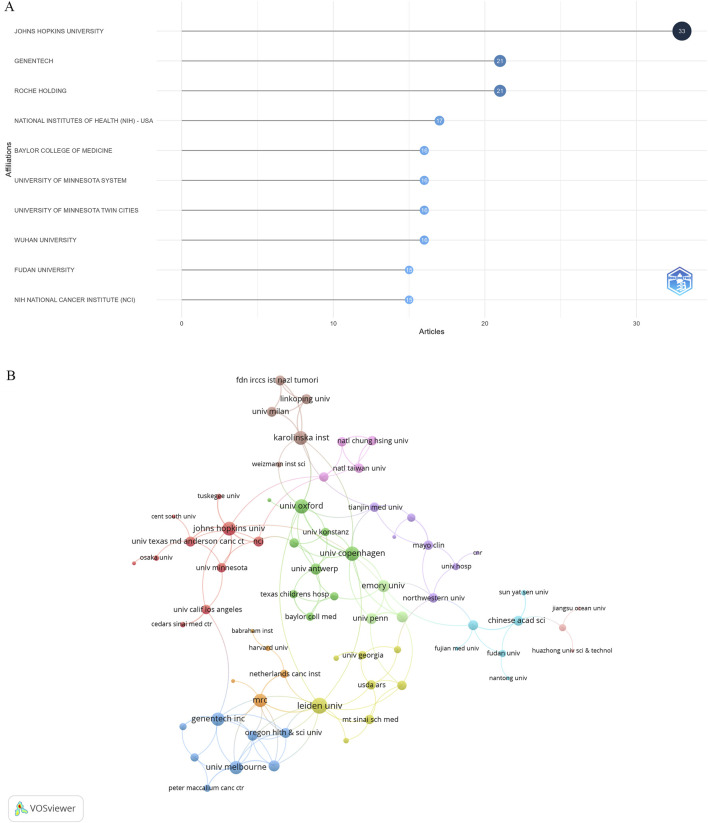
Visualization of institutions. **(A)** Publications by institution. **(B)** Collaborative networks of institutions.

Publications related to research on DUBs in ovarian cancer involved 926 institutions. The three institutions with the most publications were Johns Hopkins University (United States, 33 publications), Genentech (United States, 21 publications), and Roche Holding (United States, 21 publications) (Figure 5A). Institutions with at least two publications were included in the analysis of collaborative networks, which were visualized using VOSviewer. The clusters were arranged in different colors based on the frequency of collaboration between institutions (Figure 5B). Johns Hopkins University had the largest node, indicating the highest level of collaboration with other institutions.

### Analysis of journals and co-cited journals

**FIGURE 6:**
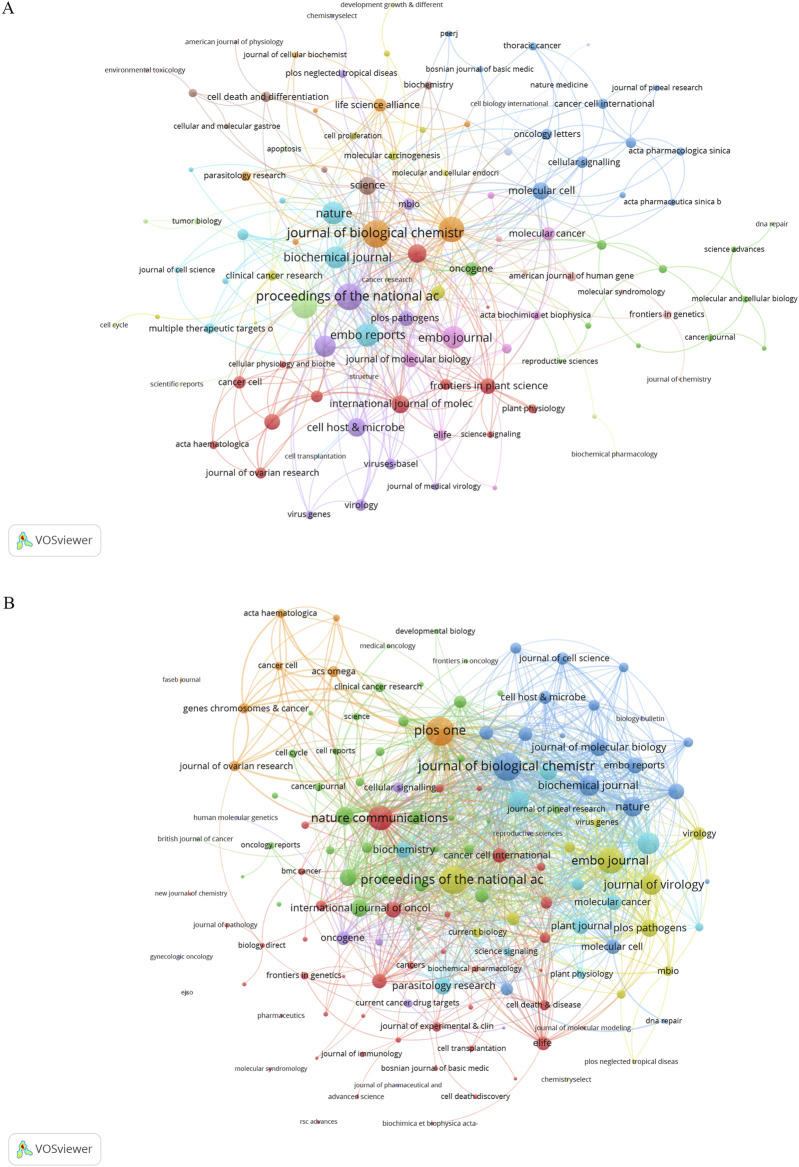
Co-citation and bibliographic coupling analysis. **(A)** Co-occurrence networks: journal link strength in co-occurrence networks measures the frequency with which two journals are cited together within the same articles or references. This metric reflects how often the publications from two different journals are associated in the bibliographies of scholarly articles. High link strength implies that the journals are often cited in tandem, indicating a thematic or topical connection between the research they publish. **(B)** Coupling networks: journal link strength in coupling networks assesses the extent to which journals are linked based on the common references cited in their articles. This metric captures the degree to which the research published in two different journals relies on the same body of prior work. Strong link strength in this context signifies that the journals share a substantial number of references, highlighting a shared intellectual foundation or research focus.

Research on DUBs in ovarian cancer prominently features in 152 journals. *Biochemical and Biophysical Research Communications* leads with nine publications, accounting for 3.70% of the total, followed by the *Journal of Biological Chemistry* and *PLOS One*, each with seven papers, accounting for 2.88% each (Table 2). Co-citation analysis revealed that the five key journals with the highest total link strength were the *Journal of Biological Chemistry* (56), *Proceedings of the National Academy of Sciences of the United States of America* (54), *PLOS One* (48), *Cell* (47), and *EMBO Reports* (40) (Figure 6A). Bibliographic coupling analysis indicated that the five key journals with the highest total link strength were *PLOS One* (1,110), *Proceedings of the National Academy of Sciences of the United States of America* (1,053), *Journal of Biological Chemistry* (1,049), *EMBO Journal* (901), and *Nature Communications* (818) (Figure 6B).

**TABLE 2:** Top 20 productive journals related to DUBs in ovarian cancer.

Journal	IF (2023)	JCR_Quartile	H_index	PY_start	TP	TP_rank	TC	TC_rank	g-index	m-index
Biochemical and Biophysical Research Communications	2.5	Q3	6	2005	9	1	59	36	9	0.300
Journal of Biological Chemistry	4	Q2	6	2003	7	2	527	1	7	0.273
Nature Communications	14.7	Q1	6	2013	6	4	153	11	6	0.500
Oncotarget	N/A	N/A	6	2014	6	5	N/A	N/A	6	0.545
Cell Death and Differentiation	13.7	Q1	5	2016	5	6	64	32	5	0.556
Journal of Virology	4	Q2	5	2010	5	8	193	8	5	0.333
PLOS One	2.9	Q1	5	2010	7	3	146	12	7	0.333
Proceedings of the National Academy of Sciences of the United States of America	9.4	Q1	5	2011	5	10	287	5	5	0.357
International Journal of Oncology	4.5	Q1	4	2004	5	7	N/A	N/A	5	0.190
Oncogene	6.9	Q1	4	1998	5	9	178	9	5	0.148
Science Advances	11.7	Q1	4	2018	5	11	N/A	N/A	5	0.571
Biochemical Journal	4.4	Q2	3	2004	4	12	86	22	4	0.143
Cell Death and Disease	8.1	Q1	3	2022	3	13	50	40	3	1.000
EMBO Journal	9.4	Q1	3	2012	3	14	230	6	3	0.231
Genes Chromosomes and Cancer	3.1	Q2	3	2008	3	15	N/A	N/A	3	0.176
Journal of Experimental and Clinical Cancer Research	11.4	Q1	3	2019	3	17	N/A	N/A	3	0.500
Molecular Cell	14.5	Q1	3	2009	3	18	383	3	3	0.188
Nature	50.5	Q1	3	2004	3	19	486	2	3	0.143
Oncology Reports	3.8	Q2	3	2015	3	20	43	50	3	0.300
Biochemistry	2.9	Q3	2	2016	2	22	N/A	N/A	2	0.222

Note(s): H_index, h-index of the journal, which measures both the productivity and citation impact of the publications; IF, impact factor, indicating the average number of citations to recent articles published in the journal; JCR_Quartile, quartile ranking of the journal in the Journal Citation Reports, indicating the journal ranking relative to others in the same field (Q1: top 25%, Q2: 25%–50%, Q3: 50%–75%, and Q4: bottom 25%); TP, total publications; TP_rank, rank of total publications; TC, total citations; TC_rank, rank of total citations; Average citations, average number of citations per publication; PY_start, publication year start, indicating the year the journal started publication; g_index, g-index of the journal, which provides more weight to highly cited articles; m_index, m-index of the journal, which is the h-index divided by the number of years since the first published paper; N/A, not applicable.

### Analysis of authors and collaborations

**FIGURE 7:**
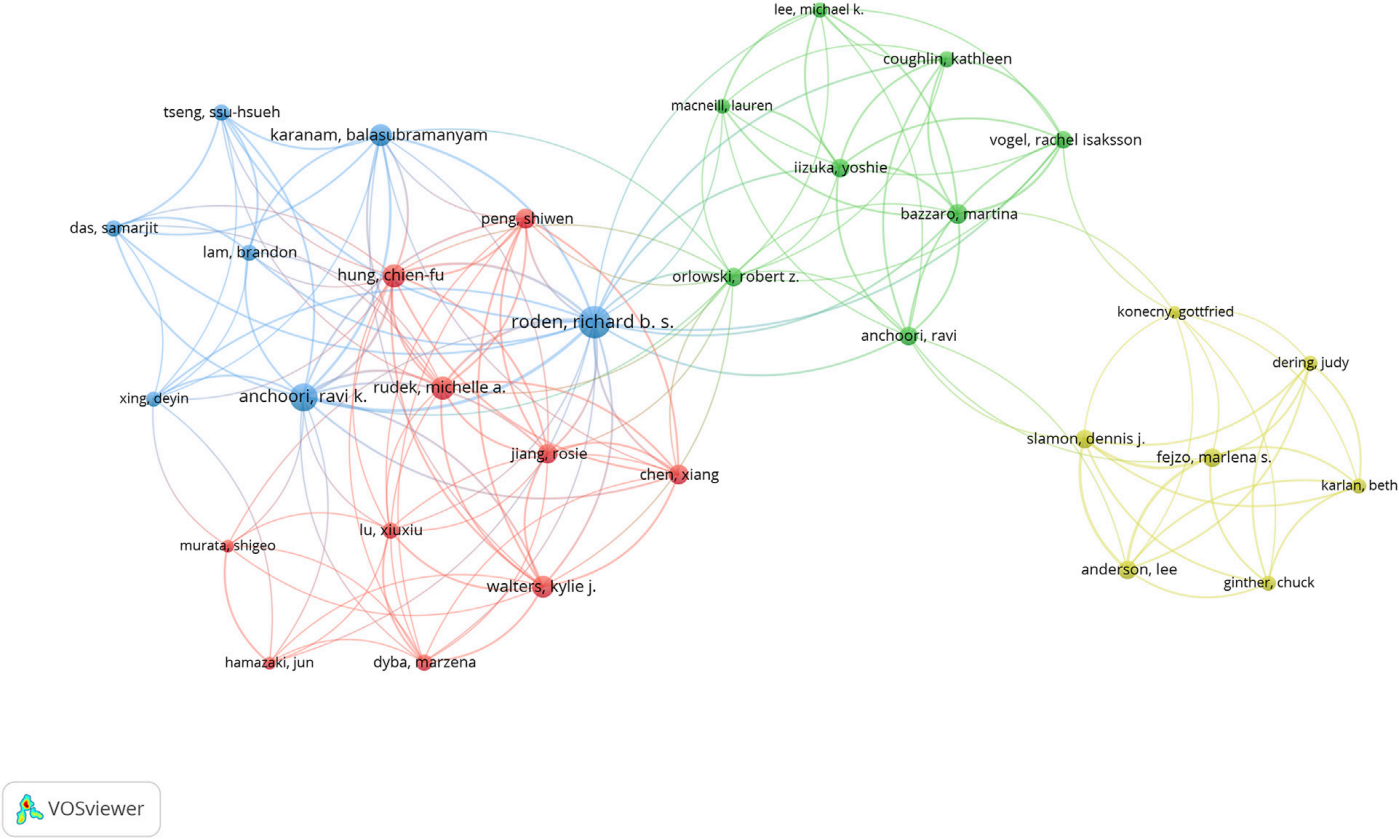
Visualization map depicting the collaboration among different authors. Nodes represent authors, with size indicating the publication count. Links represent co-authorships, with thickness showing collaboration strength. Colors indicate different research clusters. The total link strength in collaboration networks measures the frequency of co-authorship between authors, indicating the level of collaborative research.

The 243 articles were contributed by 1,895 authors. The distribution of authors was relatively concentrated, and a high degree of collaboration strength was observed. David Komander, Zhihua Liu, and Richard Roden contributed the highest number of publications, with total citations of 939, 198, and 263, respectively (Table 3). Using VOSviewer, a collaborative network analysis was conducted on authors with publication volumes of three or more. Among the 170 authors involved in international collaborations, Richard Roden had the highest number of collaborations with other countries (total link strength = 48), followed by Ravik Anchoori (total link strength = 35) and David Komander (total link strength = 27) (Figure 7).

**TABLE 3:** Publication and citation profiles of the top 20 authors.

Authors	H_index	g-index	m-index	PY_start	TP	TP_Frac	TP_rank	TC	TC_rank
Komander David	6	7	0.35	2008	7	1.20	1	939	1
Liu Zhihua	5	5	0.71	2018	5	0.74	4	198	20
Roden Richard B. S	5	7	0.42	2013	7	0.62	2	263	15
Anchoori Ravi K	4	5	0.33	2013	5	0.40	3	164	23
Anderson Lee	4	4	0.24	2008	4	0.49	6	113	33
Fejzo Marlena S	4	4	0.24	2008	4	0.49	8	113	33
Ovaa Huib	4	5	0.33	2013	5	0.41	5	599	7
Pegan Scott D	4	4	0.29	2011	4	0.65	11	116	32
Slamon Dennis J	4	4	0.24	2008	4	0.49	12	113	33
Snijder Eric J	4	4	0.22	2007	4	0.51	13	617	6
Ahel Ivan	3	3	0.75	2021	3	0.42	14	112	37
Akutsu Masato	3	3	0.21	2011	3	0.34	15	653	3
Anchoori Ravi	3	3	0.27	2014	3	0.35	16	118	31
Baek Kwang-Hyun	3	3	0.20	2010	3	0.89	17	35	46
Bazzaro Martina	3	4	0.27	2014	4	0.45	7	135	25
Bergeron Eric	3	3	0.20	2010	3	0.48	18	126	29
Ding Fang	3	3	0.43	2018	3	0.37	19	176	22
Dixit vishva M	3	3	0.17	2007	3	0.18	20	736	2
Frias-Staheli Natalia	3	3	0.17	2007	3	0.34	21	484	12
Fu Hongyong	3	4	0.27	2014	4	0.68	9	69	43

Note(s): H_index, h-index of the journal, which measures both the productivity and citation impact of the publications; g_index, g-index of the journal, which provides more weight to highly-cited articles; m_index, m-index of the journal, which is the h-index divided by the number of years since the first published paper; TP, total publications; TP_rank, rank of total publications; TC, total citations; TC_rank, rank of total citations; Average citations, average number of citations per publication; PY_start, publication year start, indicating the year the journal started publication.

### Analysis of research hotspots and frontiers

**FIGURE 8:**
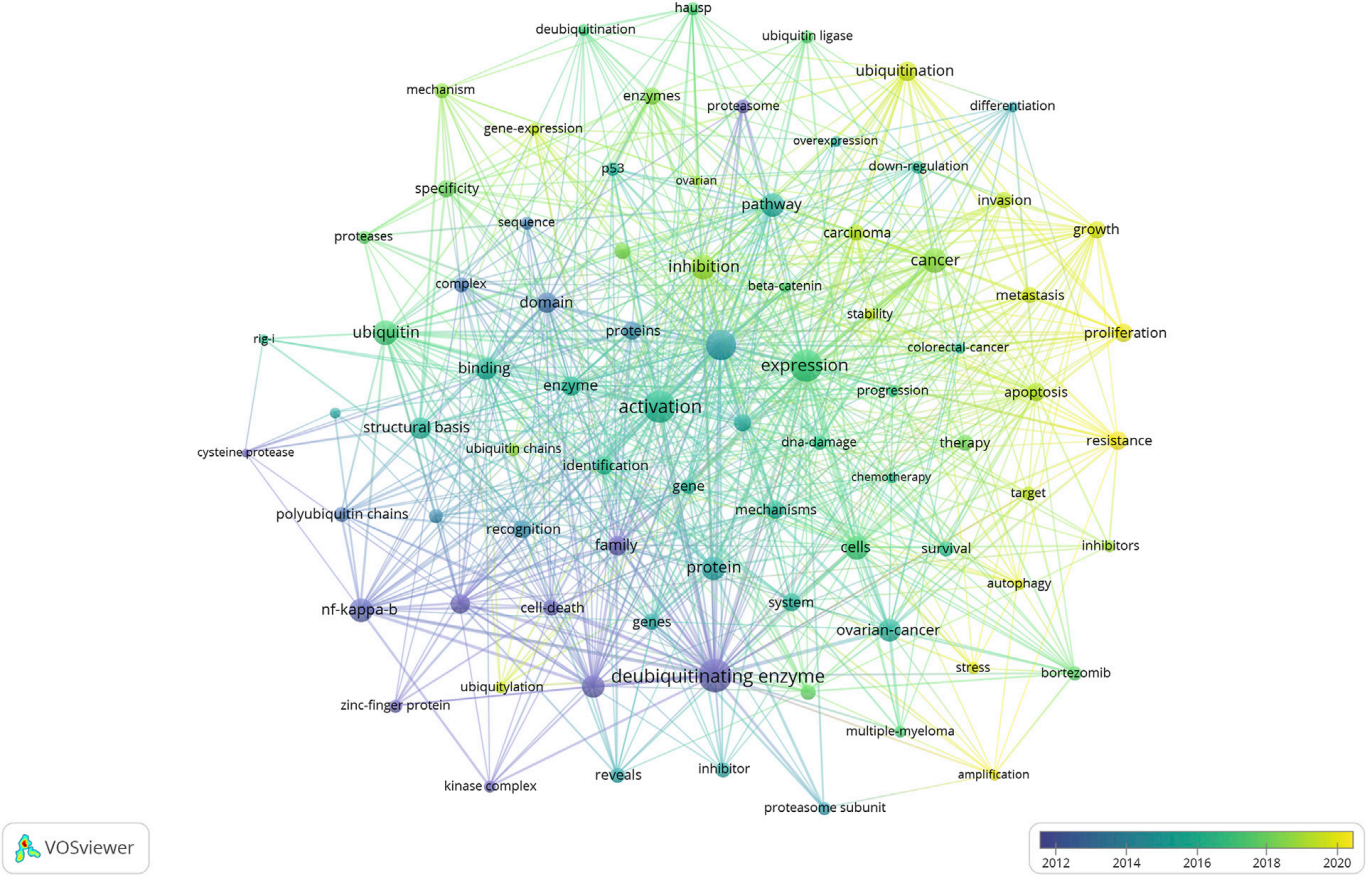
Visualization of keyword co-occurrence. This network visualization displays the co-occurrence of keywords in selected literature. Each node represents a keyword, with size indicating its frequency of occurrence. Links between nodes represent co-occurrence in the same documents, with thicker lines showing stronger associations. Colors reflect the average publication year of the articles, as indicated by the color gradient at the bottom right.

Keywords succinctly encapsulate the fundamental concepts of a paper, outlining the key areas of research interest. A comprehensive keyword analysis of the selected 243 articles related to DUBs was performed using “Author Keywords” from the Biblioshiny application and “Keywords Plus” provided by the VOSviewer application. In total, 566 keywords were identified. A network visualization map demonstrating the connections among these keyword co-occurrences was generated using VOSviewer. The sizes of the circles correspond to the frequency of occurrence of the keywords. A co-word analysis revealed that “deubiquitinating enzyme,” “degradation,” “expression,” “activation,” and “ubiquitin” were the most frequently co-occurring keywords (Figure 8). The top 20 co-occurring keywords are given in Table 4.

**TABLE 4:** Top 20 keyword co-occurrence network analysis.

id	Keyword	Occurrences	Total link strength
208	Deubiquitinating enzyme	34	136
15	Activation	35	129
304	Expression	40	127
200	Degradation	29	110
890	Ubiquitin	27	76
425	Inhibition	20	74
100	Cancer	26	74
688	Protein	24	71
129	Cells	21	69
567	nf-kappa-b	17	68
622	Pathway	19	66
595	Ovarian cancer	21	62
181	Cysteine proteases	14	62
71	Binding	14	58
812	Structural basis	17	56
235	Domain	12	54
172	Crystal structure	10	49
314	Family	11	48
910	Ubiquitination	12	48
283	Enzyme	10	47

**FIGURE 9:**
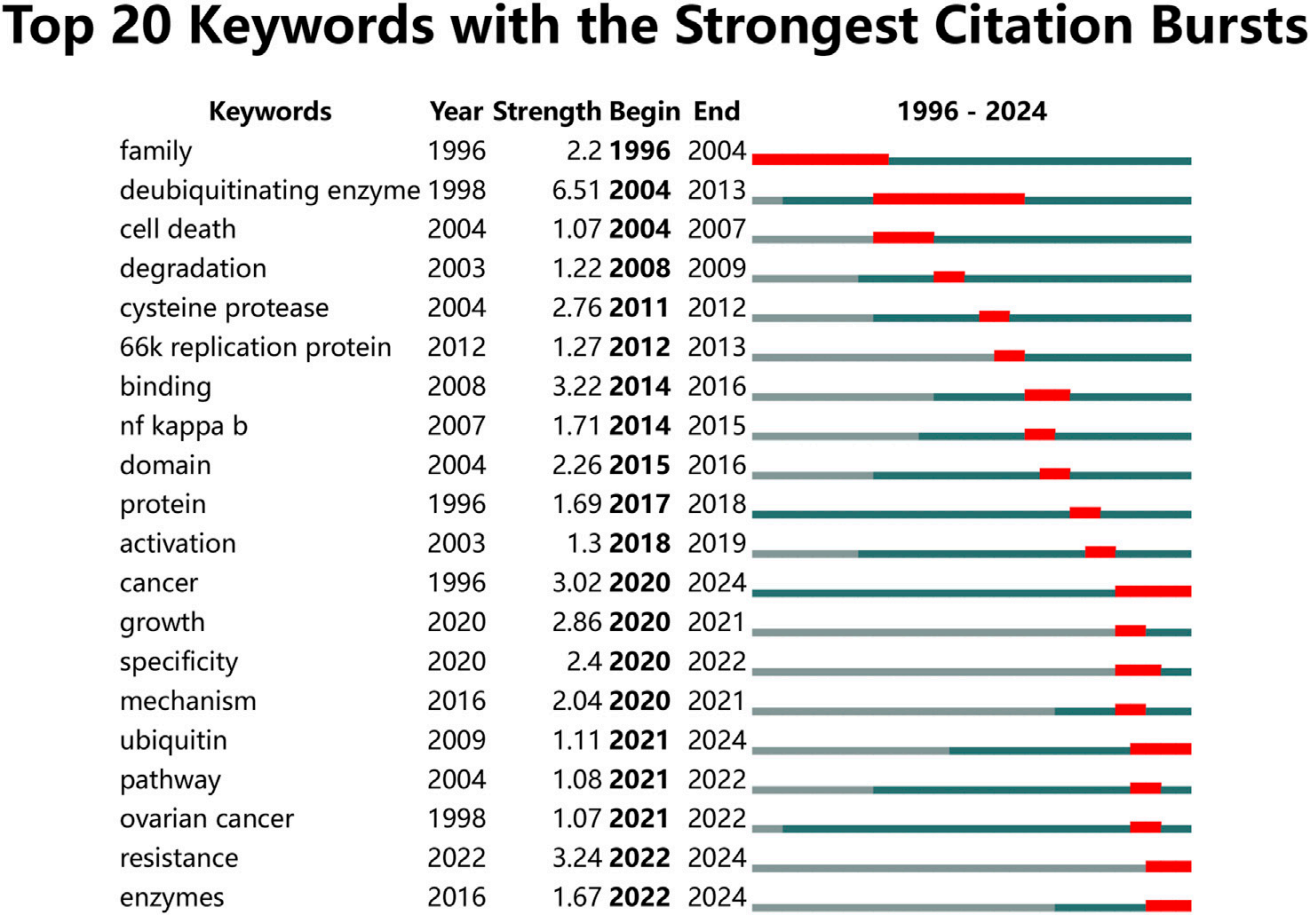
Top 20 keywords with the strongest citation bursts.

Figure 9 presents the top 20 keywords with the highest burst strengths. The most significant citation burst belongs to “deubiquitinating enzyme.” Particularly noteworthy is the concentration of keywords such as “cancer,” “growth,” “specificity,” “mechanism,” “ubiquitin,” “pathway,” “ovarian cancer,” “resistance,” and “enzymes” since 2020, indicating promising developments.

## Discussion

Since 1996, studies on DUBs in ovarian cancer have experienced rapid growth, particularly after 2002, driven by their pivotal biological roles in cancer research. It is evident that DUBs have gradually emerged as a hotspot in ovarian cancer, indicated by an average citation of 47.41 per article. Additionally, the number of articles on DUBs in ovarian cancer has steadily increased, with an annual growth rate of 8.57%. Since 2020, keyword concentrations have focused on “cancer,” “growth,” “specificity,” “mechanism,” “ubiquitin,” “pathway,” “ovarian cancer,” “resistance,” and “enzymes,” highlighting future research directions for DUBs in ovarian cancer. Additionally, the most frequently co-occurring keywords are “deubiquitinating enzyme,” “degradation,” “expression,” “activation,” and “ubiquitin,” indicating that a deep understanding of the mechanisms of DUBs in ovarian cancer is a critical medical need. Interestingly, these keywords are centered around the critical regulatory functions of DUBs, suggesting that DUBs are widely entangled with the classic signaling pathways that have been well understood in ovarian cancer development. These findings highlight that DUBs may be of equal importance as the key regulatory proteins in cell division, growth, and proliferation, encouraging research workers to include DUBs as a part of the cellular regulatory network rather than as a simple tool for protein degradation and recycling. Therefore, based on this bibliometric analysis, studies of DUBs on ovarian cancer are likely to continue advancing by understanding their roles in cancer development and their potential as therapeutic targets.

The countries with the highest publication volume are primarily China, the United States, and the United Kingdom. China ranks the first in terms of publication quantity, whereas the United States and the United Kingdom have the highest average citations (all higher than 100) and intermediary centrality, highlighting their active and prominent roles in this field. However, the average citation frequency per paper in China is low, indicating that Chinese authors have lower citation frequencies, highlighting the need of high-quality paper publication. Notably, the top three institutions contributing to the publication volume were all from the United States, indicating a pioneering role in driving DUB-related research in ovarian cancer. Johns Hopkins University, Roche Holding, and Genentech had the highest intermediary centrality, serving as crucial contributors to fundamental DUB research in this disease. The top three cited articles had 1,509, 573, and 429 citations, respectively, and were published in *Nature* (impact factor = 50.5), *Oncogene* (impact factor = 6.9), and *Cell* (impact factor = 45.5) (Wertz et al., 2004; Jensen et al., 1998; Mevissen et al., 2013). All three articles focused on the mechanism of DUBs, highlighting the critical need of the mechanical analysis of this malignant disease.

We summarized the expression profile and biological roles of DUBs in ovarian cancer. Specifically, the following terms were used for the database search without language and regional restrictions: “ovarian cancer” or “ovarian neoplasms” AND “deubiquitinating enzymes” or “deubiquitinases” or “ovarian tumor proteases” or “ubiquitin-specific proteases” or “ubiquitin C-terminal hydrolases” or “Josephin domain-containing proteins” or “motif interacting with Ubcontaining novel DUB family” or “JAB1/MPN/Mov34 metalloenzyme.” Other eligible studies were also reviewed from the references of each article. As we retrieved zero results for Josephin domain-containing proteins in ovarian cancer, we mainly focused on the expression and functional role of OTUs, USPs, and UCHs in ovarian cancer (Table 5). Research workers may utilize this information to develop treatments against important molecular targets, such as mutant p53 and PTEN, or explore DUBs as potential therapeutic targets. For instance, USP7 is one of the representative DUBs that have been widely studied in cancer research. It exerts fine-tuned control over diverse protein levels and functions, impacting cell fate decisions and maintaining cellular homeostasis. USP7 is a critical regulator of many cancer-related proteins, including p53, MDM2, PTEN, and FOXO4. Zhang et al. (2016) suggested that USP7 expression is associated with poor prognosis in ovarian cancer, supported by cellular experiments. Ma and Yu (2016) found that USP7 is highly expressed in epithelial ovarian cancer patients, positively correlated with lymphatic invasion, and independently associated with poor overall survival. They concluded that the modulation of USP7 expression could affect ovarian cancer cell viability and invasion (Ma and Yu, 2016). Wang et al. (2017) reported that the inhibition of USP7 could induce cell death in ovarian cancers, regardless of the P53 status. This finding is consistent with that of previous research, showing that USP7 was highly expressed in ovarian cancer and inversely correlated with the differentiation level, and that inhibition of USP7 could lead to cell apoptosis (Qin et al., 2016). Furthermore, Wang et al. (2023b) found that USP7 deubiquitinases TRAF4, and the knockdown of USP7 suppressed ovarian cancer both *in vitro* and *in vivo*. A recent meta-analysis concluded that USP7 promotes ovarian cancer progression and predicts unfavorable clinical outcomes (Kisaï and Koji, 2021). These findings suggest that USP7 may act as an oncoprotein highly expressed in ovarian cancer cells and patients, and may be associated with poor clinical outcomes. In addition, USP14 may be another promising target in ovarian cancer treatment, with the earliest research traced back to 2007 (Yang et al., 2007). Subsequent studies have revealed the critical involvement of USP14 in various pathways, especially in tumor proliferation and chemoresistance (Wang et al., 2015; Wada et al., 2009; Shen et al., 2020; Huang et al., 2017; Luo et al., 2019; Ji et al., 2023). It can thus be hypothesized that targeting USP14 may be an effective strategy for second- and third-line therapies, during which chemoresistance is the major challenge. Moreover, UCHL1 is another interesting target for its broad implications in various ovarian cancer cell lines, as well as animal models and patient samples (Tangri et al., 2021; Okochi-Takada et al., 2006; Jin et al., 2013). Understanding its roles in different cell lines and signaling pathways may reveal common mechanisms in ovarian cancer development. It should be emphasized that although most DUBs are not direct executers in signaling pathways, they may be equally important as they essentially modulate the concentrations of the key regulators. This can be utilized to create novel therapeutic strategies against certain oncoproteins, especially against those with various mutations or thought to be “undruggable” (Lei et al., 2021b). For example, KRAS mutation is known to promote ovarian cancer development (Therachiyil et al., 2022), yet only a few drugs are proven effective against certain mutations of KRAS. Instead of directly inhibiting KRAS, inducing KRAS degradation by activating its DUB(s) may be a promising approach; furthermore, this strategy may be a “one-size-fits-all” solution that is robust against various KRAS mutations (Fraile et al., 2017), which may also be extended to other critical targets in cancer therapy.

**TABLE 5:** Summary of DUB biological function in ovarian cancer.

Family	DUBs	Author	Year	Source	Target	Mechanism
Ovarian tumor protease (OTU)	OTUB1	Wang et al. (2016)	2016	A2780, SKOV3, CAOV3, and ovarian cancer patients	FOXM1	Tumor progression and prognosis
		Wu et al. (2021)	2021	HeLa and SW620	/	Chemoresistance
		Maresca et al. (2015)	2015	Ovarian cancer tissue	/	Tumorigenesis
	OTUD3	Johnson et al. (2020)	2020	Bioinformatics analysis, OVSAHO, PEO1, and OVCAR5	PTEN and RIPK	Necroptosis
	ALG13	Wang (2021)	2021	Bioinformatics analysis	/	Prognosis
	A20	Lin et al. (2016)	2016	SKOV3	CYLD	Chronic inflammation, apoptotic resistance, and invasion
	OTUD7A	Tavares et al. (2021)	2021	Bioinformatics analysis	/	/
Ubiquitin-specific proteases (USPs)	USP1	Sonego et al. (2019)	2019	MDAH-2774, TOV-21G, OV-90, SKOV3, OVCAR3, OVCAR4, OVCAR8, OVSAHO, KURAMOCH, and ovarian cancer tissue	Snail	Platinum resistance and metastasis
		Simoneau et al. (2023)	2023	*BRCA1/2* mutant and wild-type tumor	PCNA	Apoptosis
		Song et al. (2022)	2022	OVCAR8, EFO21, and bioinformatics analysis	S phase	Cell cycle
	USP2	Yang et al. (2007)	2007	Ovarian cancer tissue	/	/
	USP5	Du et al. (2019)	2019	Ovarian serous carcinoma specimen, OVCAR3, A2780, HO-8910, CAOV3, SKOV3, and xenograft model	HDAC2	Apoptosis
	USP7	Zhang et al. (2016)	2016	Primary serous ovarian cancer specimen and SKOV3	March7	Cell proliferation, invasion
		Ma and Yu (2016)	2016	Primary serous ovarian cancer specimen, SKOV3, and OVCAR3	/	Overall survival, lymph node metastasis, cell viability, and invasion
		Wang et al. (2017)	2017	HeyA8 and OVCAR8	/	Cell death and autophagy
		Qin et al. (2016)	2016	Ovarian cancer tissue array, SKOV3, HO-8910 OVCAR3, A2780, A2780/CP70, HeyC2, and xenograft model	Mdm2, Mdmx, and UHRF1	Cell death
		Wang et al. (2023b)	2023	Ovarian cancer tissue, CAOV-3, SKOV3, and xenograft model	TRAF4	Proliferation, migration, and invasion
		Kisaï and Koji (2021)	2021	Meta-analysis	/	Cancer progression and prognosis
	USP8	Corno et al. (2022)	2022	IGROV-1, A2780, PEO1, PEO4, PEO6, IGROV-1/Pt1, A2780/CP, A2780/BBR, and advanced ovarian cancer patients	/	Drug resistance and apoptosis
	USP9X	Hunter et al. (2015)	2015	Low-grade serous ovarian tumor specimen	/	Tumorigenesis
		Habata et al. (2016)	2016	AMOC2, ES2, and primary ovarian cancer specimens	Mcl-1	Chemoresistance
	USP10	Han et al. (2019)	2019	Epithelial ovarian cancer tissue microarray	/	Prognosis
		Gao et al. (2022)	2022	Bioinformatics analysis	Immune infiltration	Prognosis
		Li et al. (2022a)	2022	Ovarian cancer tissue array, OVCAR3, ES2, A2780, SKOV3, and IGROV1	G3BP1	Cancer progression and metastasis
	USP11	Wang et al. (2019a)	2019	Ovarian cancer tissues, OVCAR-3, and SKOV3	Snail	Epithelial-to-mesenchymal transition
		Zhu et al. (2021)	2021	Ovarian cancer specimen, ES2, and 3AO	BIP	Chemoresistance
		Guo et al. (2022) and Stiff et al. (1994)	2022, 1994	Refractory ovarian cancer patients	/	/
	USP13	Han et al. (2016)	2016	Ovarian cancer specimens, CAOV3, OVCAR3, HeyA8, OVCAR8, and SKOV3	PIK3CA	Cancer metabolism
		Zhang et al. (2018)	2018	SW-1573, TOV-21G, xenograft model, and ovarian cancer specimen	MCL1	Proliferation
		Li et al. (2017)	2017	OVCAR3, SKOV3, A2780, FU-OV-1, EFO-27, and xenograft model	RAP80-BRCA1	DNA damage
		Kwon et al. (2022a)	2022	Xenograft model and primary ovarian specimen	/	Cancer development and metastasis
		Kwon et al. (2022b)	2022	HeyA8 and COV318	/	Proliferation
	USP14	Yang et al. (2007)	2007	Ovarian cancer tissue	/	/
		Wang et al. (2015)	2015	Epithelial ovarian cancer tissue and SKOV3	/	Proliferation, prognosis
		Wada et al. (2009)	2009	SHIN-3	/	Tumorigenesis
		Shen et al. (2020)	2020	A2780, COC1, A2780/CP, and COC1/CP	BCL6	Chemoresistance
		Huang et al. (2017)	2017	A2780, SKOV3, and xenograft model	/	Proliferation and tumor growth
		Luo et al. (2019)	2019	A2780 and A2780/CDDP	Connexin 32	Chemoresistance
		Ji et al. (2023)	2023	A2780, OVCAR8	BACH1	Heme metabolism and invasion
	USP15	Xu et al. (2009)	2009	HeLa	Caspase-3	Apoptosis
		Eichhorn et al. (2012)	2012	/	TβR-I	Tumorigenesis
		Padmanabhan et al. (2018)	2018	SKOV3, SK-BR-3, YK-Nu, OVCAR3, OVCA420, S1GODL, MDAH2774, COV362, and TOV-112D	p53-R175H	Cell death
	USP17	Yildirim et al. (2019)	2019	High-grade, advanced-staged serous ovarian cancer biopsy	/	Epithelial-to-mesenchymal transition
	USP18	Liu et al. (2022)	2022	A2780, SKOV3, and bioinformatics analysis	AKT/mTOR	Proliferation and migration
		Li et al. (2022b)	2022	A2780 and OVCAR8	FBXO6	Tumorigenesis
	USP19	Kang et al. (2021)	2021	Advanced-stage high-grade serous ovarian carcinoma specimen	/	Prognosis
	USP22	Ji et al. (2015)	2015	SKOV3, OVCAR3, epithelial ovarian cancer specimen, and xenograft model	TGFβ1	Proliferation, prognosis and cell cycle
		Gennaro et al. (2018)	2018	/	/	Tumorigenesis, cell cycle
	USP28	Ito et al. (2018)	2018	TU-OC-1, KOC7c, RMG-1, RMG-2, TOV-21G, ES2, and SKOV-3	Claspin	Cell viability
		Shen et al. (2023)	2023	OVCAR3, A2780, and ovarian cancer patients	β-catenin	Proliferation
		Aziz et al. (2018)	2018	High-grade serous ovarian cancer specimens	Cyclin E1	Prognosis
	USP32	Nakae et al. (2021)	2021	SKOV3, OVCAR3, A2780, high-grade serous ovarian cancer specimen, and xenograft model	FDFT1	Progression and prognosis
	USP34	Zhao et al. (2023)	2023	Bioinformatics analysis	/	Prognosis and immune microenvironment
	USP35	Zhang et al. (2021)	2021	Ovarian cancer tissue, VCAR3, SKOV3, VCAR-5, ID8, and xenograft model	STING	Prognosis, immune infiltration, and chemoresistance
	USP36	Li et al. (2008)	2008	A2780, Caov-3, and ovarian cancer tissue	/	/
		Yan et al. (2020)	2020	OVCAR8, SKOV3, OV-90, OVCAR10, IGROV1, OVKATE, OV-56, PEO1, and ovarian cancer specimen	PrimPol	DNA replication and chemoresistance
	USP39	Wang et al. (2021)	2021	Primary ovarian cancer patients, A2780, SKOV3, OVCAR3, OVCAR8, CAOV3, ID8, and xenograft model	HMGA2	Malignancy
		Wang et al. (2019b)	2019	SKOV3, ES2, and xenograft model	/	Malignancy and chemoresistance
		Yan et al. (2019)	2019	HO8910, SKOV3, and xenograft model	p53/p21	Proliferation and epithelial-to-mesenchymal transition
	USP44	Lu et al. (2014)	2014	T80 and SKOV3ip1	/	Cell cycle progression and proliferation
		Tserpeli et al. (2021)	2021	Advanced high-grade serous ovarian cancer	/	/
	USP45	Liu et al. (2023b)	2023	SKOV3, OVCAR3, serous ovarian cancer specimen, and xenograft model	Snail	Tumorigenesis, progression, and chemoresistance
	USP46	Xu et al. (2021)	2021	Ovarian cancer specimen, SKOV3, and SKOV3/DDP	Bcl-2/caspase-3 and ATK	Proliferation, apoptosis, and chemoresistance
	USP47	Hu et al. (2019)	2019	SKOV3, TOV-112D, and ovarian cancer specimen	/	Proliferation
	USP48	Lei et al. (2020)	2020	ES2, 3AO, A2780, ovarian cancer specimen, and xenograft model	/	Chemoresistance and metastasis
	USP51	Zou et al. (2015)	2015	Bioinformatics analysis, SKOV3, SKOV3/DDP, A2780, and A2780/DDP	/	/
Ubiquitin C-terminal hydrolases (UCHs)	UCHL1	Tangri et al. (2021)	2021	Bioinformatics analysis, high-grade serous ovarian cancer patient specimens, xenograft model, OVCAR4, COV362, OVCAR8, OVCAR3, SKOV3, A2780, and HeyA8	PSMA7-APEH-proteasome	Proliferation, invasion, survival, and tumor growth
		Okochi-Takada et al. (2006)	2006	OV90, MCAS, RMUG-L, RMG-I, RTSG, TYK-nu, TOV112D, ES2, HTOA, KURAMOCHI, JHOS-2, and TOV-21G	/	/
		Jin et al. (2013)	2013	A2780, A2780CP, SKOV3, IGROV1, ES2, OVCAR3, and CAOV3	BCL2, BCL11A, AEN, and XIAP	Proliferation, cell cycle, and chemoresistance
		Gutkin et al. (2019)	2019	High-grade serous ovarian cancer patient specimen	/	Tumorigenesis and immunogenicity
		Alur et al. (2019)	2019	Bioinformatics analysis	/	Progression
	UCHL3	Li and Wang (2019)	2019	SKOV3 and IGROV1	/	Progression
		Zhang et al. (2020)	2020	Bioinformatics analysis, xenograft model, SKOV3, ES2, HO8910, A2780, and COC1	TRAF2	Proliferation, migration, and inflammatory response
	UCHL5	Wang et al. (2014b)	2014	Epithelial ovarian cancer specimen	/	Tumor progression and prognosis
		Fukui et al. (2019)	2019	Tissue microarray, MESOV, SKOV3, OVISE, RMG-1, ES2, and xenograft model	Smad2	Progression-free survival and apoptosis
		Huang et al. (2017)	2017	A2780, SKOV3, and xenograft model	/	Proliferation and tumor growth
	BAP1	Devins et al. (2023)	2023	Ovarian low-grade serous carcinoma specimen	/	/
		Chapel et al. (2017)	2017	Ovarian serous tumor specimen	/	/
		Wang et al. (2022c)	2022	Bioinformatics analysis	/	/
		Chui and Grisham (2023)	2023	Ovarian serous borderline tumor and recurrent low-grade serous carcinoma specimen	/	/
		Davidson et al. (2018)	2018	Ovarian serous tumor specimen	/	/

Keywords reflect the primary content of publications and encapsulate the main topics covered in the literature. Analyzing keywords can offer insights into current study hotspots and future directions in the research field. By examining the frequency and co-occurrence of keywords, research workers can identify prevailing themes and emerging trends that shape the field trajectory. In this study, “deubiquitinating enzyme,” “degradation,” “expression,” and “activation” were the most frequently co-occurring keywords. These keywords highlight the central themes of current research, emphasizing the role of DUBs in cellular processes. DUBs are known for their ability to remove ubiquitin from target proteins, thereby preventing their degradation. This stabilization affects the activation and localization of various proteins, triggering cascades of biological processes that are crucial for maintaining cellular homeostasis and function. A timeline viewer for keyword analysis reveals the evolution of hotspots in the field over time, showing how the focus within the field has shifted and expanded. This tool helps visualize the progression of key research topics and provides a historical perspective on how the field has developed. For instance, the consistent appearance of terms like “degradation,” “expression,” and “activation” underscores the ongoing interest in understanding the fundamental mechanisms of DUBs and their broader biological implications. Regarding keywords with the strongest citation bursts, “cancer,” “ubiquitin,” “resistance,” and “enzymes” have been the latest hotspots in ovarian cancer research since 2020, and the focus on “ubiquitin” and “resistance” as future directions highlights the need for more research into how ubiquitin signaling pathways contribute to cancer progression and treatment outcomes. Understanding these pathways could lead to the development of novel interventions that target specific DUBs or their substrates, potentially overcoming resistance to current therapies and improving patient outcomes.

This bibliometric analysis provides a comprehensive and visual analysis of DUBs in ovarian cancer; however, several limitations should be acknowledged. This study only included articles indexed in the WoSCC, and the language was restricted to English. Therefore, publications in other databases or languages were not included in the analysis. Nevertheless, the WoSCC is a well-recognized database, and given its prominence, the impact of such omissions on the overall findings is expected to be low. Further studies are needed to include additional databases and languages to provide a more accurate and comprehensive analysis. Based on the narrative review and the bibliometric analysis, future studies may need to focus on the potential of DUBs as drug targets for the treatment and management of this disease.

## Conclusion and outlook

In summary, a visual analysis of DUBs is presented in this study in the field of ovarian cancer research, facilitated by the use of CiteSpace, VOSviewer, and R4.3.3. The essential functions of DUBs in ovarian cancer biology include DNA repair, cell cycle regulation, apoptosis, oncogenic signaling, chemotherapy response, and chemoresistance. However, the precise functions and mechanisms of DUBs in ovarian cancer remain largely unexplored. Moreover, the expression levels and functions of some DUBs are still under debate; whether these DUBs serve as oncogenic proteins, tumor suppressors, or double-edged swords in ovarian cancer requires further investigation. Understanding the intricate interplay between DUBs and ovarian cancer biology offers promising prospects for developing innovative and more effective treatment strategies, ultimately improving outcomes for patients with this challenging disease. Future efforts are expected to decipher the specific roles of individual DUBs in ovarian cancer, identify potential therapeutic targets, and explore the feasibility of targeting DUBs as a novel approach to treating ovarian cancer.

## Data Availability

The original contributions presented in the study are included in the article/Supplementary Material; further inquiries can be directed to the corresponding authors.
