# Supplementary material for: Mapping and visualization of global research progress on deubiquitinases in ovarian cancer: a bibliometric analysis
Source: Front Pharmacol. 2024 Sep 12;15:1445037. doi: 10.3389/fphar.2024.1445037 (PMC11424541; doi:10.3389/fphar.2024.1445037)
Supplement: Supplementary file 1 [file Table1.DOCX]

**Supplementary Table 1 Search strategy**

| # | **Search strategy** | **Results** |
| --- | --- | --- |
| 1 | TS=(deubiquitin* AND enzyme*) | 5000 |
| 2 | ALL=((DUBs OR DUB) AND enzyme*) | 1611 |
| 3 | ALL=("deubiquitylating enzyme*") | 459 |
| 4 | ALL=(((USPs OR USP? OR USP-*) AND enzyme*) OR "ubiquitin-specific protease*") | 3676 |
| 5 | ALL=(((UCHs OR UCH? OR UCH-*) AND enzyme*) OR "ubiquitin C-terminal hydrolase*") | 1028 |
| 6 | ALL=(((OTUs OR OTU? OR OTU-*) AND enzyme*) OR ("ovarian tumor protease*")) | 605 |
| 7 | ALL=(((MINDYs OR MINDY? OR MINDY-*) AND enzyme*) OR ("motif interacting with Ubcontaining novel DUB family")) | 37 |
| 8 | ALL=(((Josephins OR Josephin? OR Josephin-*) AND enzyme*) OR ("Josephin domain-containing protein*")) | 544 |
| 9 | ALL=(((JAMMs OR JAMM? OR JAMM-*) AND enzyme*) OR "JAB1/MPN/Mov34") | 1256 |
| 10 | #1 OR #2 OR #3 OR #4 OR #5 OR #6 OR #7 OR #8 OR #9 | 10613 |
| 11 | TS=(ovarian cancer OR ovarian neoplasms) | 141359 |
| 12 | ALL=((neoplasms OR cancer OR carcinoma OR tumor OR malignancy) AND (ovarian OR ovary OR oophor*)) | 190276 |
| 13 | #11 OR #12 | 190276 |
| 14 | #10 AND #13 | 280 |
| 15 | #10 AND #13 and Article | 243 |
| 16 | #10 AND #13 and Article and English | 243 |
